# Performance Evaluation of Deep CNN-Based Crack Detection and Localization Techniques for Concrete Structures

**DOI:** 10.3390/s21051688

**Published:** 2021-03-01

**Authors:** Luqman Ali, Fady Alnajjar, Hamad Al Jassmi, Munkhjargal Gocho, Wasif Khan, M. Adel Serhani

**Affiliations:** 1 Department of Computer Science and Software Engineering, College of Information Technology, UAEU, Al Ain 15551, United Arab Emirates; 201990024@uaeu.ac.ae (L.A.); mgochoo@uaeu.ac.ae (M.G.); 201990025@uaeu.ac.ae (W.K.); serhanim@uaeu.ac.ae (M.A.S.); 2 Department of Civil Engineering, College of Engineering, UAEU, Al Ain 15551, United Arab Emirates; h.aljasmi@uaeu.ac.ae

**Keywords:** automatic inspection, convolutional neural networks, crack detection, deep learning, transfer learning

## Abstract

This paper proposes a customized convolutional neural network for crack detection in concrete structures. The proposed method is compared to four existing deep learning methods based on training data size, data heterogeneity, network complexity, and the number of epochs. The performance of the proposed convolutional neural network (CNN) model is evaluated and compared to pretrained networks, i.e., the VGG-16, VGG-19, ResNet-50, and Inception V3 models, on eight datasets of different sizes, created from two public datasets. For each model, the evaluation considered computational time, crack localization results, and classification measures, e.g., accuracy, precision, recall, and F1-score. Experimental results demonstrated that training data size and heterogeneity among data samples significantly affect model performance. All models demonstrated promising performance on a limited number of diverse training data; however, increasing the training data size and reducing diversity reduced generalization performance, and led to overfitting. The proposed customized CNN and VGG-16 models outperformed the other methods in terms of classification, localization, and computational time on a small amount of data, and the results indicate that these two models demonstrate superior crack detection and localization for concrete structures.

## 1. Introduction

In civil infrastructure, cracks are the earliest sign of the structural deterioration that reduces the lifespan and reliability of concrete structures, and can lead to serious environmental damage. Assessment and monitoring of the structures is required for lifetime maintenance and failure prediction. Concrete structure condition information can be obtained manually, i.e., subjective visual inspection and assessment by human experts, or automatically. Manual inspection techniques are labor-intensive, time-consuming, and inspector dependent, i.e., vulnerable to the inspector’s level of perceptiveness. Automatic inspection techniques offer an efficient solution, which eliminates subjectivity and addresses other problems associated with manual inspection.

Over the last decade, advances in computer science have enabled the use of vision-based machine learning and deep learning approaches to autonomous civil infrastructure inspection. The development of an effective vision-based automated concrete crack detection system has been a challenging research problem. These systems rely heavily on images of the structure. Such images are often affected by various factors, such as low contrast between the concrete surface and cracks, irregular size and random shape of cracks, intensity variations, multiple textures, and shadowing. However, these systems can detect, localize, and even provide orientation and width information of the cracks in the images. Researchers extensively studied the development of vision-based approaches for concrete crack detection, and have proposed several widely used approaches. Early image processing methods for crack detection include edge detection [1], thresholding and segmentation [2], region growing [3], and peculation-based techniques [4]. Local information-based models use various filters, such as morphological [5], statistical [6], 2D matched [7], and median filters, as well as multi-scale line filters based on the Hessian matrix [8]. Developments in machine learning have significantly influenced and improved concrete crack detection. Differing from previous approaches, machine learning can learn deep features and perform statistical inference without manually tuning parameters.

Traditional machine learning approaches for crack detection primarily comprise two steps: feature extraction, and classification. In the feature extraction step, image processing techniques are used to extract relevant crack information (features) from the images that are then evaluated by various classifiers. Many crack detection studies have been based on traditional machine learning approaches [9,10,11,12,13,14,15]. However, if the extracted features do not reflect the actual cracks, the classifier may not provide accurate results. Deep learning methods have significantly extended the versatility and robustness of conventional approaches, and have shown great performance in solving crack detection problems [15,16,17,18,19,20,21,22,23,24]. Deep convolutional neural network (CNN) models are capable of extracting relevant features from the input data through a multi-layer neural network [25]. Moreover, these models are also extendable to perform crack localization by using the sliding window approach. In the sliding window approach, a bounding box is drawn on the patch location classified as a crack by the classifier. To construct a deep learning model, the correct network structure, loss function, and an effective optimization algorithm must be selected. 

Zhang et al. [26] proposed a six-layer convolutional neural network (CNN) architecture for crack detection in pavement surfaces, and used 640 k, 160 k, and 200 k images to train, validate, and test the network, respectively. Wang et al. [27] proposed a CNN architecture with three convolutional and two fully connected layers for asphalt pavement crack recognition. The authors used 640 k image patches to train, and 120 k patches to test, the model. In [28] the authors combined CNN architecture with sliding window techniques for crack detection and localization of cracks in concrete images using 40 images. Based on their results, the authors recommended using more than 10 k images to train a network from scratch. Xu et al. [29] trained a 28-layer end-to-end CNN model for concrete bridge crack detection using 6069 images acquired from a bridge structure. The authors used the concept of atrous spatial pyramid pooling (ASPP) to gain multiscale context information and depth-wise convolution to reduce the number of network parameters. In [30] the authors proposed a CNN model for crack detection in pavement structures, to investigate the effect of network depth and image position on the performance of the model. The results show that the network could be enhanced by increasing the network depth while the network generalization ability decreased with changing the position of training and testing images. Fan et al. [31] proposed an efficient automatic pavement crack detection and measurement model using an ensemble of CNN models. The authors used a weighted average ensemble technique to calculate the final crack probability by combining the probability score from the individual CNN models. In [32], Tong et al. designed three different (recognition, location, and feature extraction) CNN architectures with different functionalities to perform crack detection, localization, length measurement, and 3D reconstruction of concealed cracks in ground penetrating radar (GPR) images. Yang et al. [33] proposed a novel approach for automatic detection and measurement of pixel-level cracked concrete structures using a deep learning approach, named fully convolutional network (FCN). The architecture was composed of down sampling (conventional CNN layers) and up sampling (deconvolutional layer). Zhang et al. [34] proposed CrackNet, a CNN architecture consisting of five (2 convolutional, 2 fully connected, and 1 output) layers for pixel-level crack detection on 3D asphalt surfaces. CrackNet could efficiently detect cracks at pixel level, but the processing time of the architecture was high, and it had difficulty in detecting hairline cracks. To overcome these problems, the authors proposed CrackNet II [35] to improve the processing time of the network, eliminate local noises, and detect hairline cracks in 3D asphalt surfaces. The above-mentioned networks were trained from scratch, and require a large amount of training data and considerable time. Training time can be minimized by fine-tuning pretrained models that have been used for a similar task. 

Transfer learning models facilitate the applicability of CNNs without incurring high computational costs or requiring knowledge about how CNN’s operate. Transfer learning models that use image data as input include Google models, i.e., the Visual Geometry Group’s VGGNet [36], Microsoft’s ResNet [37] and Inception-V3 [38]. Gopalakrishnan et al. [39] used pre-trained VGG-16 architecture for pavement crack detection. Here, the authors used a comparatively small size of data, i.e., training data, 760 images; validation data, 84 images; and test data, 212 images. Compared to previous CNN models, the proposed transfer learning model was reliable, fast, and easily implementable. Zhang and Cheng [40] used an ImageNet-based pre-trained model to identify cracks and seal cracks in pavement images. In that study, the training dataset consisted of 30 k crack patches, and 30 k non-crack patches. In the test set, 20 k patches were assigned to each class. In [41] the authors used the VGGNet model with 2000 labeled images (4:1 training to test data) to detect various types of structural damage. Bang et al. [42] trained a deep residual network with transfer learning for road crack detection using 118 images. Wilson and Diogo [43] performed robust training of the VGG-16 model for concrete crack detection using 3.5 k unmanned aerial vehicle (UAV) acquired crack and non-crack images; here, the data was split 80/20 for training and test data. Gopalakrishnan et al. [44] proposed an automatic crack detection system based on the VGG-16 model using images acquired by a drone. Feng and Zhang [45] modified the architecture of the Inception V3 model, and trained it using transfer learning to detect structural damage in concrete water pipes.

All the above-mentioned deep learning approaches have shown promising performance for crack detection problems which motivated us to explore the performance of different state-of-the-art DCNN architectures, focusing on the significance of data design in the architecture. The previous studies were performed by considering various factors, e.g., data selection, filter choice, and the number of layers used in the architecture of the models. However, no information has been provided about the effect of data size, variance in the data, the number of required epochs, and network depth on the performance of these architectures. It is difficult for the researchers working in the field to select an appropriate deep learning architecture and dataset size for their problems. The main advantage of selecting an appropriate deep learning architecture and data size is that it ensures better generalization of the model and prevents it from overfitting. Moreover, by keeping a balance between the amount of training data, complexity of the model, and number of model parameters, the computational and time complexity of the model is also reduced. Hence, we herein present a study to evaluate the performance of five deep neural network architectures for crack detection and localization in concrete structures. The evaluation of each model is based on how the number of training samples, diversity in training samples, number of epochs, network complexity, number of layers, and parameter tuning affect the performance of these models. The evaluation metrics include the accuracy, precision, recall, receiver operating characteristic (ROC) curve, and localization results of the models. The main contributions of the proposed research work are the following:

1. We proposed a customized CNN architecture for crack detection and localization in concrete structures. The proposed model was compared with various existing models based on various factors, e.g., training data size, heterogeneity among the data samples, computational time, and number of epochs, and the results demonstrate that the customized CNN model achieved a good balance between accuracy, network complexity, and training time. The results also show that a promising level of accuracy can be achieved by reducing data collection efforts and optimizing the model’s computational complexity.
2. We investigated the effect of network complexity, data size, and variance among data samples on the performance of the models. The results clearly show that network complexity and variance in the data sample have the greatest influence on the model performance and are more important than the size of the data.
3. Based on the experimental results, a discussion was undertaken which provides the significance of the deep learning models for crack detection in a concrete structure. In general, the discussion provides a reference for researchers working in the field of crack detection and localization using deep learning techniques.

The remainder of this paper is organized as follows. Section 2 provides an overview of the proposed system. The CNN and various pre-trained models are described in Section 3, and Section 4 discusses our experiments and presents the experimental results, and an overall discussion is given in Section 5. Finally, conclusions are presented in Section 6.

## 2. Overview of the Proposed System

**Figure 1:**
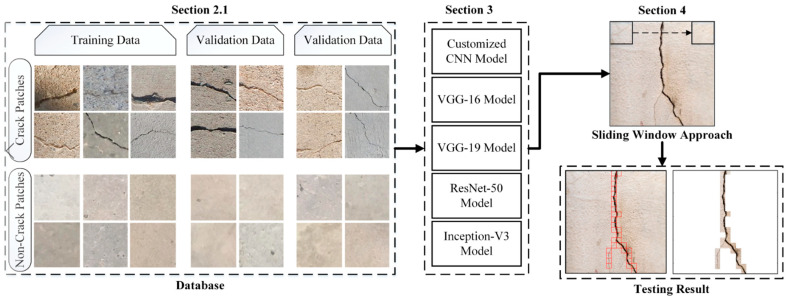
Outline of proposed work.

A flowchart of the proposed system is shown in Figure 1. The first module represents the database preparation step, and the second module shows the architecture and implementation of deep learning algorithms for crack detection in concrete structures. The evaluation and comparison of the models based on various metrics are explained in the last module.

### Dataset Preparation

In the proposed system, the dataset was created from publicly available datasets by [46,47]. The size and variance in a dataset have a significant effect on the generalization of deep learning models [48]. Therefore, to provide heterogeneity or variance among the data samples, the above-mentioned two datasets were combined, and a base image classification dataset of 25 k images was created. The data samples from both datasets were selected equally, with the ratio of 0.5, and the variance was provided based on the type of concrete surface, illumination shadowing, and crack patterns. In addition, seven image classification datasets containing 20.8 k, 15.6 k, 13.4 k, 10.4 k, 8.4 k, 5.6 k, and 2.0 k images with a resolution of 224 × 224 were created from the base dataset (Table 1). The patches were selected randomly from the datasets, and the split ratio for the training, validation, and testing sets was 60:20:20. Manual labeling was performed for crack and non-crack classes, where each class contained an equal number of image patches (Figure 1). The idea behind the creation of various datasets was to evaluate how model performance varies with changes in dataset size

## 3. Training Models

The performance of five CNN models, i.e., the customized CNN, VGG-16, VGG-19, ResNet-50, and Inception-v3, was evaluated. All the pretrained models were trained on the ImageNet dataset, and each model is explained in detail below.

### 3.1. Customized CNN Model

**Figure 2:**
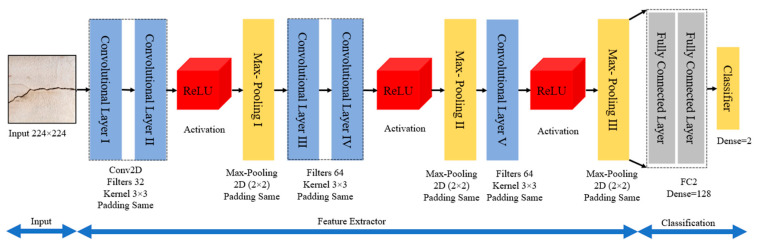
Convolutional neural network (CNN) architecture.

In the proposed work, a CNN architecture was constructed from scratch by fine-tuning various hyperparameters in the architecture, such as the number of convolutional and fully connected layers, the number of filters, stride, pooling locations, sizes, and the number of units in fully connected layers. The selection of hyperparameters was performed manually on a trial-and-error basis, as there is no mathematical formulation for the calculation of appropriate parameters for a specific dataset. The overall computational architecture of the proposed customized CNN is shown in Figure 2. The architecture consists of five convolutional, three activation, three max-pooling, and two fully connected layers, and a softmax layer. The main role of these layers is to increase model performance by extracting useful features, reducing data dimensionality, and introducing nonlinearity [49]. The convolutional layers were used in blocks to improve the spatial invariance property, which helps in the recognition of important features in the input crack images. A CNN architecture depends on spatial or sequential features of the data to learn. If the input data to the network is highly sparse, the learning ability of the network is highly degraded. Solutions for this problem have been reported in the available literature [50,51]. However, in the proposed work, an Adam optimizer was used as an adaptive learning-rate method to handle sparse input data [52]. Although RMSprop, Adadelta, and Adam are very similar algorithms, the idea of using Adam was that it performs well at the end of optimization as the gradients become sparser.

Here, the first layer takes an image of h×w×c pixels as input and passes it through various convolutional and max pooling layers to reduce its spatial size. (where h, w, and c represent the image height, width, and the number of channels). After passing through several convolutional and max-pooling layers, the final feature vector is obtained at the fully connected layer, and is input into the classifier for class prediction.

#### 3.1.1. Convolutional Layer

The convolutional layer’s main task is to extract relevant features by finding local connections among the data samples coming from the input layer (Equation (1)).

(1) $$Feature\;Vector=\;\sum^{\;}\left(Input_{k\times k}+Weights_{k\times k}\right)+B\;$$

Here, $Input_{k\times k}$ represents the input local receptive field on which the convolutional operation is performed. $Weights_{k\times k}$, k, and b are filter weights, kernel size, and filter bias of the convolutional layer operation, respectively. In the convolutional layer, the feature vector is obtained by convolving the filter over the image pixels, and adding the pixels together. Note that the obtained feature maps vary with different convolutional kernels. The final feature vector obtained is then inputted into the activation layer (ReLU).

#### 3.1.2. Activation Layer

**Figure 3:**
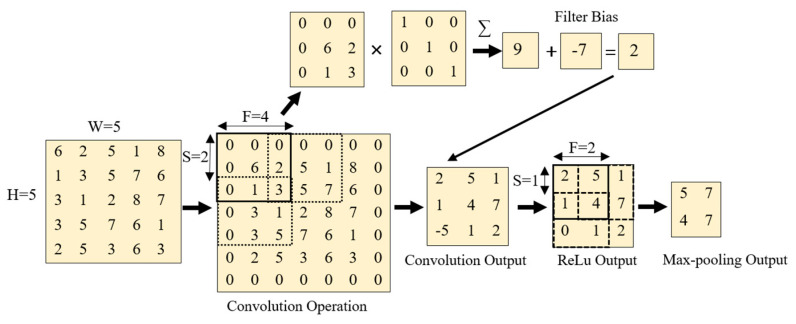
Mathematical operations in convolutional, rectified linear unit (ReLu), and max-pooling layers.

The rectified linear unit (ReLU) was introduced by Nair et al. [53]. The ReLU sets the nonnegative values obtained from the convolutional layer to zero, by performing an elementwise operation (Equation (2)). This layer guarantees the usability of the feature maps obtained from the convolutional layer by introducing nonlinearity. The activation layer (i.e., the ReLU) is commonly used due to its faster computational capabilities than other activation functions, e.g., sigmoid and tanh. The mathematical operations of the activation layer are shown in Figure 3.

(2) σ(I)=max(0,I)

Here, I represents the elements in the input vector.

#### 3.1.3. Max-Pooling Layer

The max-pooling layer divides the prior feature map into small nonoverlapping pooling kernels. Here, the maximum value of each kernel is considered and forwarded to the next layer. The main tasks performed in the max-pooling layers are: (1) down sampling the data obtained from the previous layer to reduce the dimensionality of the data; and (2) reducing the number of model parameters, reducing computational time, and improving the model generalizability.

#### 3.1.4. Fully Connected Layer

The fully connected layer can be considered a traditional neural network that performs logical inference. In the proposed system, the fully connected layer converts the three-dimensional matrix obtained from the previous layers to a one-dimensional vector using a full convolution operation. The mathematical operation of the fully connected layer is expressed as follows.

(3) $$Z_{V_{o}\times 1}=Weight_{V_{o}\times V_{i}}\;I_{V_{i}\times 1}\cdot \;Bias_{V_{o}\times 1}$$

Here, Vi and Vo represent the input and output vector size, and Z represents the output of the fully connected layer. Moreover, the weights and bias matrix are represented by Weight and Bias, respectively. 

#### 3.1.5. Softmax Layer

The final layer in the CNN architecture is the softmax layer, which is used to calculate normalized class probabilities $P\left(y^{\left(i\right)}=n^{\left(i\right)}|x^{\left(i\right)};W\right)$ for each class $n^{\left(i\right)}$, in n classes, using Equation (4).

(4) $$\left(y^{\left(i\right)}=n^{\left(i\right)}|x^{\left(i\right)};W\right)=\left[\begin{array}{c} y^{\left(i\right)}=1|x^{\left(i\right)};W\\ y^{\left(i\right)}=2|x^{\left(i\right)};W\\ \vdots \\ y^{\left(i\right)}=n|x^{\left(i\right)};W \end{array}\right]=\frac{1}{\sum_{j=1}^{n}e^{W_{j}^{T}x^{\left(i\right)}}}\left[\begin{array}{c} e^{W_{1}^{T}x^{\left(i\right)}}\\ e^{W_{2}^{T}x^{\left(i\right)}}\\ \vdots \\ e^{W_{n}^{T}x^{\left(i\right)}} \end{array}\right]$$

Here, m is the total number of data samples, where i=1…m. W represents the weights, and the input to the classifier is denoted $e^{W_{n}^{T}x^{\left(i\right)}}$. Equation (4) takes a vector with arbitrary real-valued scores as input, and outputs a vector with values between 0 and 1.

### 3.2. Pre-Trained VGG-16 Model

**Figure 4:**
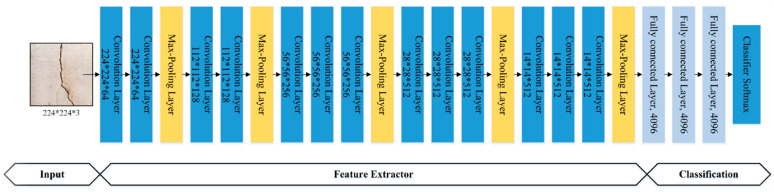
The architecture of the VGG-16 model.

VGG-16 is an open-source CNN model proposed by [36]. This model was submitted to the ImageNet Large Scale Visual Recognition Challenge (ILSVRC-2014). The VGG-16 model comprises 16 layers (13 convolutional layers and three fully connected layers with a filter size of 3 × 3), as shown in Figure 4. The 13 convolutional layers are divided into five groups, and a max-pooling layer follows each group. The input to the VGG-16 architecture is a 224 × 224-pixel RGB image. The input passes through a stack of convolutional and max-pooling layers, and, in the end, a (7, 7, 512) feature map is received, which is then flattened into a (1, 25088) size feature vector. The flattened feature is then input to the three fully connected layers with the same configurations, and a (14,096) feature vector is obtained. The output of the fully connected layers is then inputted into the softmax layer for classification.

### 3.3. Pre-Trained VGG-19 Model

**Figure 5:**
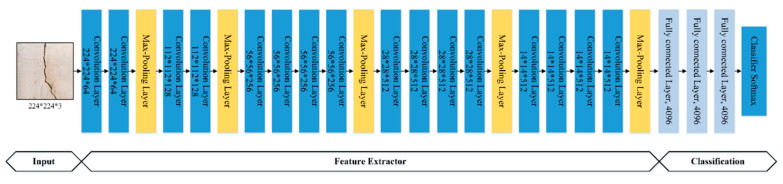
The architecture of the VGG-19 model.

The VGG-19 transfer learning model was proposed by [36]. This model comprises 19 layers (16 convolutional and three fully connected layers with a filter size of 3 × 3, and a stride and pad size of 1 pixel). The small kernel size reduces the number of parameters, and enables them to cover the entire image. In the VGG-19 model, a 2 × 2 max pooling operation with stride 2 is performed. This model ranked second in classification and first in positioning at the 2014 ILSVRC, with a total of 138 million parameters. VGGNet reinforced the concept that CNNs must have a deep layer network, such that visual data can be interpreted hierarchically. The block model of VGG-19 is shown in Figure 5.

### 3.4. ResNet-50 Model

**Figure 6:**
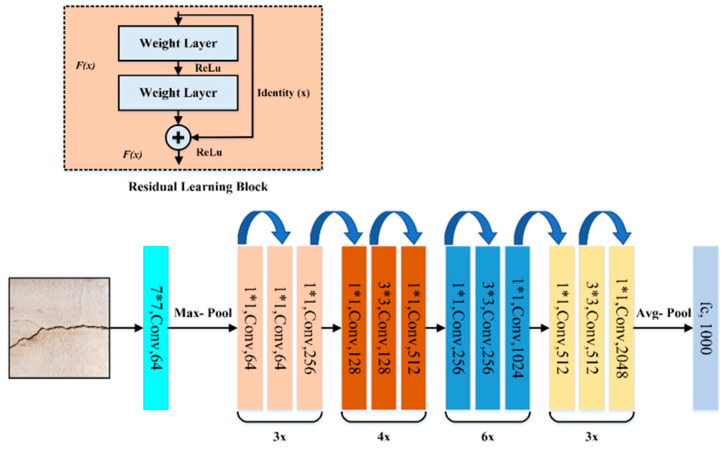
The architecture of ResNet-50 model.

He et al. [37] proposed the residual neural network (ResNet), which took first place at the ImageNet Large Scale Visual Recognition Challenge (ILSVRC 2015). ResNet introduced residual connections between layers, which helps reduce loss, preserve knowledge gain, and boosts performance during the training phase. A residual connection in a layer means that the output of a layer is a convolution of its input plus its input. A block diagram of the ResNet model’s architecture is shown in Figure 6. 

### 3.5. Inception-V3 Model

**Figure 7:**
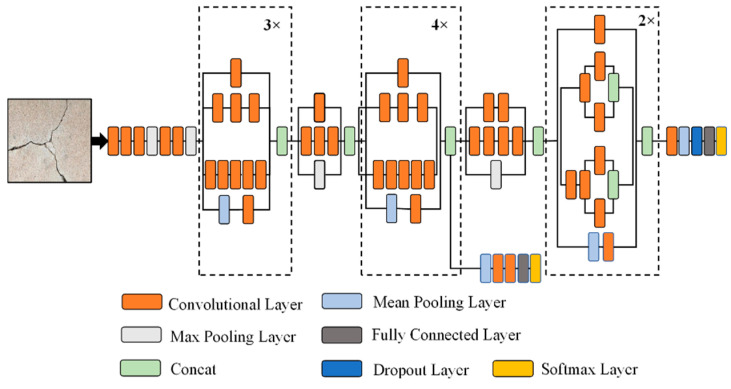
The architecture of Inception-V3 model.

The Inception-V3 pre-trained model was proposed by [38]. This model comprises over 20 million parameters, and has been trained by one of the industry’s top hardware experts. The model itself comprises symmetrical and asymmetrical building blocks, where each block consists of various convolutional, average, and max pooling, concats, dropouts, and fully connected layers. In addition, batch normalization is commonly used and applied to the activation layer input into this model. Classification is performed using Softmax. A schematic diagram of the Inception-V3 model is shown in Figure 7.

## 4. Experiments and Results

**Figure 8:**
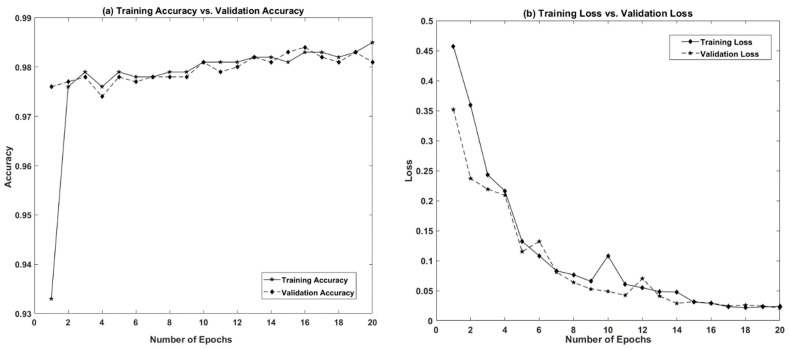
Training and validation of the proposed model (2.8 k Dataset). (**a**) Accuracy graph. (**b**) Loss graph.

The performance of five deep learning models for concrete crack detection was evaluated based on input data size, model complexity, convergence rate, accuracy, precision, recall, and F1 score. The experiments were conducted using eight datasets of different sizes (25 k, 20.8 k, 15.6 k, 13.4 k, 10.4 k, 8.4 k, 5.6 k, and 2.8 k (as shown in Table 1)) made from two publicly available datasets. The experiments were conducted using Python programming on an Alienware Arura R8 core i9-9900k CPU @3.60 GHz desktop system with 32 GB RAM and an NVIDIA GeForce RTX 2080 GPU. The number of convolutional layers and the parameters of each model are shown in Table 2. It can be seen from the table that the proposed CNN model possesses a minimal number of convolutional layers and network parameters compared with the other pretrained models. The VGG models are three to four times deeper than the proposed model, and have 53 times more network parameters than the proposed model. The architecture of ResNet is like VGG models, but deeper than VGG models. The complexity of the ResNet model is lower than VGG models in terms of its number of parameters. VGG models have a five-times greater number of parameters than ResNet 50. Moreover, the depth and number of network parameters of the ResNet-50 and Inception-V3 models are approximately the same. In the previous CNN-based crack detection approaches [26,46,54,55,56] various numbers of epochs have been taken during the training phase, ranging from 10 to 100. In the proposed work, the number of epochs in the experiments for all the models (either trained from scratch or fine-tuned from pre-trained models) was chosen as 20. The loss of the models reached a minimum level at the 20th epoch, and there was no further increase in the accuracy of the models, as shown in Figure 8. The minimum number of epochs helps in reducing the computational time of the model [57]. Moreover, a greater number of epochs may sometimes lead to overfitting of the models [58]. 

### 4.1. Evaluation Metrics

In the proposed work, various evaluation metrics such as accuracy, precision, recall, and F1 score were considered for a fair comparison of the experimental results. Accuracy can be defined as the ratio of correctly identified crack and non-crack patches to the total number of input patches, as shown in Equation (5).

(5) $$Accuracy\;=\;\frac{TP+TN}{TP+FP+TN+FN}$$

where *TP* (True Positive) and *TN* (True Negative) represent the correctly identified crack and non-crack patches, while *FP* (False Positive) and *FN* (False Negative) represent the incorrectly identified crack and non-crack patches. Precision is the ratio of correctly identified crack patches and the total number of crack patches identified by the classifier as shown in Equation (6).

(6) $$Precision=\;\frac{TP}{TP+FP}$$

Recall can be defined as the number of correctly identified crack patches and the total number of crack patches as depicted in Equation (7).

(7) $$Recall=\;\frac{TP}{TP+FN}$$

Similarly, the *F*1 score is defined as the harmonic mean of the model’s precision and recall. The *F*1 score can be formulated in Equation (8):

(8) $$F_{1\;score}=2\times \frac{Precision\cdot Recall}{Precision+Recall}$$

### 4.2. Classification Results

The experiments were conducted by comparing the results obtained by the customized CNN, VGG-16, VGG-19, ResNet-50, and Inception-V3 models. Note that five models were trained with eight different varying datasets for 20 epochs, and a total of 8000 trained networks were obtained. For simplicity, the best performing trained network for each model was selected, based on its accuracy, precision, recall and F_1_ score, as shown in Table 3. The performance of each model was also evaluated by giving a new set of test images. For all 40 trained networks, it was evident from the results that the performance metrics of all models were compatible with each other. The accuracy, precision, recall, and F1 score of all models were greater than 0.90. The customized CNN model, VGG-16, and Inception-V3 model performed well with smaller datasets, while the VGG-19 model benefited from larger datasets. The ResNet-50 and Inception-V3 models demonstrated a slight change in performance after increasing the size of the training data. The accuracy at the first and twentieth epochs for all models is summarized in Table 4 to provide insight into the convergence rate of each model. All models demonstrated fast convergence at a smaller numbers of epochs (except ResNet-50, which required a high number of epochs to achieve better test results compared to the other models).

**Figure 9:**
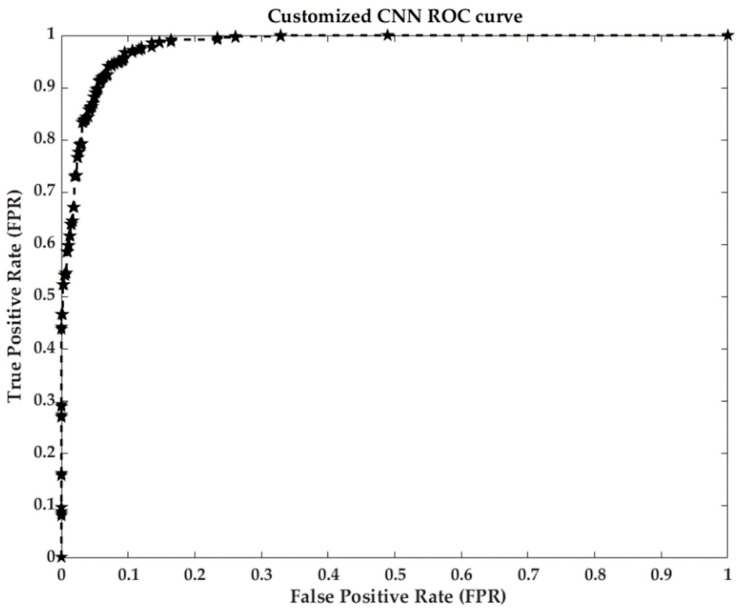
Receiver Operating Characteristic (ROC) curve of the proposed Customized CNN model.

The customized CNN model performed well, and achieve an accuracy of greater than 0.95 on all datasets. However, the performance of this model decreased gradually with increasing data size. The best validation and testing scores (0.99 and 0.98, respectively) were obtained when using a 2.8 k dataset. The accuracy and loss graphs (training and validation) of the proposed model are shown in Figure 8, while a ROC curve is shown in Figure 9. There was slight divergence between the training and validation accuracy of the proposed model, which shows that the model was not subjected to overfitting. We found that the validation and testing score of the model decreased slightly with an increasing training data size. Using a 25 k dataset, training and testing scores of 0.96 and 0.95 were achieved, respectively. When the model was trained using a small amount of training data, the model converged faster at lower epochs and did not require higher epochs to achieve the best score. Moreover, the performance of this model decreased gradually and was subject to overfitting as the size of the training data increased from 8.4 k to 10.4 k, 13.4 k, 15.6 k, 20.8 k, and 25 k. The higher accuracy and stability of the proposed model on smaller dataset sizes and a lower number of epochs shows the significance of hyperparameter optimization for the model. The model skipped unwanted features at lower epochs due to the use of an activation function along with a dropout function.

The VGG-16 model obtained better performance relative to accuracy, precision, recall, and F1 score for all training data sizes. However, there was a barely noticeable decrease in the validation and testing accuracy as the training data size increased. The convergence rate of the VGG-16 model was significant at lower epochs compared to the other trained networks. The best training and validation accuracies (0.99 and 0.99, respectively) obtained by the VGG-16 model were achieved using a data size of 13.4 k. VGG-16 outperformed all 40 trained networks. Overall, the accuracy and loss curves (training and validation) for the VGG-16 model did not demonstrate divergence, which indicates that this model was not subject to overfitting. Additionally, as shown in Table 3, the VGG-16 model demonstrated better performance relative to accuracy, precision, recall, and F1 score for all training data sizes. This feature learned by the VGG-16 can be easily transferred to other materials at high performance.

The VGG-19 did not outperform the customized CNN and VGG-16 models with a small number of training samples. The model showed a tendency to overfit with the 2.8 k dataset, as the model is deeper than VGG-16. As the size of the training data increased, the performance of the model increased gradually, and the best performance was achieved using larger datasets. The best validation and testing accuracy (0.95 and 0.95, respectively) was achieved using a dataset containing 25 k images. The model VGG-19 converged faster with a larger dataset, and required more epochs to achieve better performance for larger datasets. As shown in Table 4, the accuracy of the VGG-19 model differed noticeably at the first and twentieth epochs.

The training and testing accuracies of the ResNet-50 model in all scenarios were promising, regardless of the training data size. However, the convergence rate of the ResNet-50 was less than that of the VGG-16 and Inception-V3 models. This model obtained a low score at the first epoch and achieved a prominent test score at the 20th epoch. The ResNet-50 model also demonstrated a tendency to overfit on smaller datasets, e.g., 2.8 k, 5.6 k, and 8.4 k, at earlier epochs. The best testing accuracy obtained by the ResNet-50 model (0.99) was obtained using data sizes of 13.4 k, 15.6 k, and 25 k. As shown in Table 4, it converged faster on larger datasets and obtained a high-test score of 0.9974 at the 20th epoch. The ResNet-50 model was not able to obtain a high score at lower epochs, due to the concept of identity mapping, which make the architecture more complex. 

With the Inception-V3 model, the best validation and testing scores (0.99 and 0.99, respectively) were obtained using a dataset size of 8.4 k. By increasing the data size, the accuracy and loss (training and accuracy) of this model experienced divergence, which indicates that this model is subject to overfitting with larger datasets. In addition, the Inception-V3 model did not require a high number of epochs to achieve high test scores. In addition to the size of the training data, variance in the training data also had a significant effect on the performance of all the models. In most of the models, such as the Customized CNN model, VGG-16, Resnet-50, and Inception-V3, the smaller dataset had sufficient variance amongst samples, and achieved the best score as compared to larger datasets. An increase in the number of samples from a single concrete structure keeps the factors such as concrete surface, intensity variation, shadowing the same, so increasing data size will just increase the computational time. The models analyzed similar features at every epoch, which minimized the test score of the system. The average time required by the models for classification of a single patch is summarized in Table 5.

For the customized CNN, VGG-16, VGG-19, and ResNet-50 models, a 2240 × 2240-pixel test image was taken. This image had 100 patches of size 224 × 224. With the Inception-V3 model, a test image of size 2290 × 2290 was selected, and this image also contained 100 patches of 229 × 229 size. The computational time required to process the test images for all models was divided by 100 to calculate the computational time for a single patch. The customized CNN model performed the fastest, requiring 0.0048 s to classify a 224 × 224 image. The time required by the VGG-16 model to classify a single image patch was 0.1995 s, which is approximately 40 times faster than that of the customized CNN model. By comparing both models, the proposed CNN model was 40 times faster than the VGG-16 model. The VGG-19 model obtained a single patch classification time of 0.2093 s, which is similar to the VGG-16 model. The time required by the ResNet-50 model for single patch testing was 0.0662 s, which is longer than the Inception V3 and customized CNN models, but less than the VGG-16 and VGG-19 models. Similarly, the single patch classification time of the Inception-V3 model was greater than the customized CNN, but less than that of the VGG-16, VGG-19, and ResNet-50 models. The VGG-19 model required the most time to classify a single 224 × 224 patch, i.e., 0.2093 s. Overall, the inference time of the entire image for the proposed CNN model was 0.48 s, while for the state-of-the-art VGG-16, VGG-19, Resnet-50, and inception-V3 the entire image classification times were 19.95, 20.93, 6.62, and 3.85 s, respectively. The proposed method achieved the lowest inference time in comparison to the other methods. Additionally, the proposed method had the smallest number of model parameters, computational time, and complexity among all the models. The model size of the proposed method is ten times smaller than the ResNet-50 and Inception-V3 models. Moreover, the proposed method is 50 times smaller in model size than the VGG-16 and VGG-19 models.

The proposed work was compared with other CNN-based state-of-the-art methods [26,46,54,55,56] based on the datasets SDNET [46] and CCIC [47], which were also used in the proposed work. Table 6 clearly shows that the proposed work outperformed the existing approaches in terms of accuracy, precision, recall, and F1 score. In [26], the authors trained a CNN consisting of four convolutional and two fully connected layers by using the CICC dataset (1000 k images) and achieved precision, recall, and F1 scores of 0.8696, 0.9251, and 0.8965, respectively. In [46,56], the author trained an Alex Net architecture on 56 k and 18 k images and achieved an accuracy of 0.9045 for the bridge deck, 0.8745 for walls, 0.9486 for pavement, and 0.97 for concrete structures, respectively. Słoński et al. [54] used part of the SDNET dataset (5.4 k images) and achieved an accuracy of 0.85 by training a CNN from scratch, consisting of four convolutional and three fully connected layers. Fang et al. [55] first trained a DCNN by using SDNET and CCIC datasets, and then retrained the architecture by using a self-created dataset of 184 k images to achieve precision, recall, and F1 scores of 0.184, 0.943, and 0.307, respectively. On the other hand, the proposed model utilizes a subset of CCIC and SDNET (8.4 k images), and achieved accuracy, precision, recall, and F1 scoreof 0.980, 0.990, 0.971, and 0.981, respectively. The proposed model achieved promising results on a 25 k dataset in comparison with the state-of-the-art approaches.

### 4.3. Localization Results

**Figure 10:**
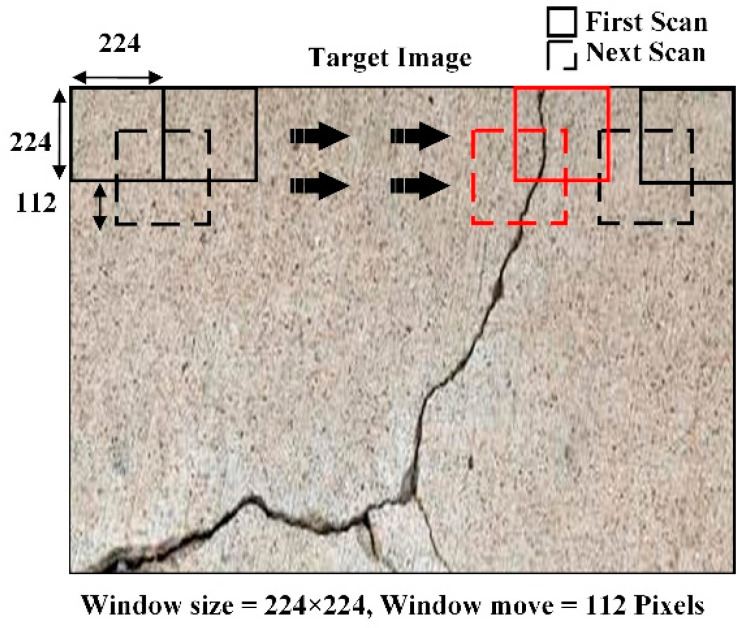
Representation of sliding window approach.

**Figure 11:**
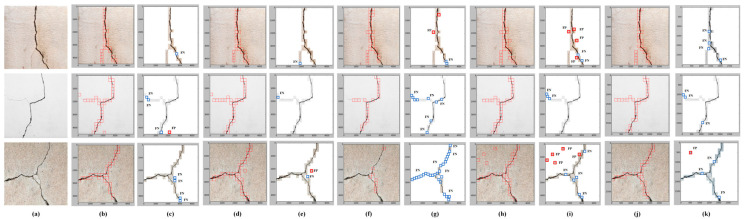
(**a**) Original images. (**b**) Crack localization using VGG-16. (**c**) Screening for false positives (FP) and false negatives (FN) using VGG-16. (**d**) Crack Localization using VGG-19. (**e**) Screening for FP and FN using VGG-19. (**f**) Crack localization using ResNet-50. (**g**) Screening for FP and FN using ResNet-50. (**h**) Crack localization using Inception-V3. (**i**) Screening for FP and FN using Inception-V3. (**j**) Crack localization using CNN. (**k**) Screening for FP and FN using CNN (8.4 k Dataset).

In this study, crack localization was performed using the sliding window technique, as shown in Figure 10. The crack localization results of all the models used in the proposed work are shown in Figure 10. The model’s crack localization performance was tested by inputting a new set of images not used in the training and validation processes. The test images were taken from various online sources. Figure 11a shows the original images input to the models for crack localization, and Figure 11b,d,f,h,j show the crack localization results obtained by the CNN, VGG-16, VGG-19, ResNet-50, and Inception-V3 models, respectively. Crack localization was performed using the sliding window approach, where a window size of 224 × 224 was used for customized CNN, VGG-16, VGG-19, and ResNet-50 models, while it was 229 × 229 for the Inception-V3 model. The window slid over the test image to perform crack localization, and the patch was classified by the trained models. The window moved by 112 pixels from left to right and top to bottom until the entire image was scanned (Figure 10). In Figure 10, the patch classified as a crack is highlighted by the red box on the borders of the patch. The part of the image inside the red bounding boxes represents the crack region. For all test images, most of the crack regions were correctly localized. The scanning results for false positives (FP) and false-negative (FN) for different images obtained by the customized CNN, VGG-16, VGG-19, ResNet-50, and Inception-V3 models are shown in Figure 11c,e,g,i,k, respectively.

The dark blue boxes represent the FN, and red boxes represent the FP regions. Each box in the localization represents a single patch in the test images. We found that the number of FP and FN patches varied in the localization results of all models. The proposed CNN model and VGG-16 models outperformed the other models in terms of localization, as the number of FP and FN patches was less than the other models. The crack localization performance of the customized CNN model was comparable with VGG-16. However, customized CNN could localize cracks in the test images five times faster than the VGG-16 model. In the VGG-16 model, the number of FP patches was greater than the number of FN patches. The FP patches mostly occurred at the edges of the image and regions that resembled a crack. With the proposed CNN model, both FP and FN patches were present. The FN patches were present in hairline crack regions, where the crack was very thin, and FP patches primarily occurred at the corner of the test image. The number of FP and FN patches in VGG-19 was greater than all the models, which is also evident from the classification accuracy of the model.

## 5. Discussion

This paper evaluated the performance of five deep learning models based on various factors, e.g., training data size, heterogeneity among data samples, number of epochs required to achieve desired test scores, and network complexity. We found that the models’ performance with a small amount of training data was comparable to the performance obtained on larger datasets. It was also found that variance among training data samples is directly related to model performance, and has a greater importance than data size. In addition, it is evident (Table 3) that the models can achieve high test scores on small training datasets if enough variance is provided among the data samples used for model training. Note that most of the deep learning models, e.g., the customized CNN, VGG-16, VGG-19, and Inception-V3 models, converged faster at earlier epochs, and did not require more epochs to obtain high test scores. However, the ResNet-50 model achieve the best performance at higher epochs. It was found that network complexity has the most significant influence on model accuracy because the best accuracy was obtained using the customized CNN and VGG-16 models, which have five and 16 convolutional layers, respectively. This is less than the number of convolutional layers in the other compared models, which reduces the computational and operational time of the proposed model. Note that the proposed model can learn features using a limited number of convolutional layers, as compared to the other pretrained models.

In addition, the Inception V3 and Resnet 50 models have a greater number of convolutional layers, and these models are also subject to overfitting. The number of parameters affects the classification time of each model, i.e., more parameters result in increased classification time (and vice versa). The customized CNN model showed the lowest patch classification time, while the VGG-19 model demonstrated the highest single patch classification time. However, the number of parameters had no impact on model performance. The results also showed that the customized CNN and Inception V-3 models tended to overfit when the number of training samples was increased gradually from 8.4 k. In contrast, the VGG-19 and ResNet-50 models suffered overfitting on smaller datasets (2.8 k, 5.6 k, and 8.4 k) at earlier epochs. Nevertheless, the results reveal that using the proposed CNN model with the sliding window technique for crack detection in concrete structures guarantees high performance. All the models showed promising performance when the dataset size was 8.4 k. Therefore, 8.4 k is sufficient for learning the dataset features. However, the optimum size of the training dataset depends on the testing images and image capturing conditions.

The proposed method enables automatic crack detection, which is very useful when inspecting concrete structures. The proposed research also demonstrates the usefulness of various deep learning models in inspecting concrete surfaces. These models ensure frequent and automatic inspection of concrete structures by providing condition information from the data they stores. In general, the proposed work provides a reference for researchers working in the field of crack detection using deep learning techniques to achieve a promising level of accuracy by reducing data collection efforts, and optimizing the model’s computational complexity. The main advantage of the proposed system is its ability to automatically detect and localize cracks using a small amount of input data, and with the minimum computation. The system can be updated by providing additional data from different structures, which can be used to detect several other types of defects in concrete structures. Despite these advantages, the proposed system is not capable of detecting and localizing cracks in real time; however, we consider that the proposed model can be used as a prototype for a real-time robotic video inspection system by integrating it with IoT devices [59]. Moreover, the proposed system is not able to analyze various characteristic of cracks, such as crack width, length, and orientation.

## 6. Conclusions

In this paper, the performance of five deep learning models, including a proposed customized CNN model, were evaluated for crack detection and localization in concrete structures using eight datasets. The dataset was generated from two publicly available datasets. Experiments were conducted by gradually increasing various factors, e.g., training data size, number of epochs, parameters, and network complexity, to investigate their effect on the performance of the compared models. It can be concluded that there is a direct relationship between model accuracy and the number of samples (training size) used for network training. The customized CNN and VGG-16 model required an optimum amount of training samples with significant variations (based on type of concrete surface, illumination shadowing, and crack patterns). However, increasing the number of training samples without sufficient variation degraded the performance of both the models, as similar image features are analyzed at each epoch. It can also be concluded that promising levels of accuracy can be achieved by using a smaller number of epochs and an optimum number of convolutional layers, by fine tuning hyperparameters of the models for the crack classification task. The number of learnable parameters had a significant effect on the computational time of the models.

The customized CNN and pre-trained models (e.g., VGG-16) can be used for automatic concrete crack detection and localization. The performance of both the models was comparable, however, the computational time and complexity of the proposed CNN model was less than the VGG-16 model. The proposed model showed better accuracy in the training, validation, and testing phases, and the features learned by this model guarantee high performance. It can also be concluded that increasing the number of layers, parameters, and training samples with minimum heterogeneity did not have a significant effect on the crack detection performance of the proposed model. However, these factors increase computational time and cause model overfitting. In conclusion, training a customized CNN model with a small amount of data and high performance is the best option for practical crack detection in concrete surfaces. In the future, we plan to explore new strategies [60] for the development of a concrete crack geometry and width estimation system at a pixel level.

## Figures and Tables

**Table 1:** Number of training, validation, and testing images in all datasets.

Dataset	Training Data		Validation Data		Testing Data	
	Crack Patches	Non-Crack Patches	Crack Patches	Non-Crack Patches	Crack Patches	Non-Crack Patches
2.8 k	840	840	280	280	280	280
5.6 k	1680	1680	560	560	560	560
8.4 k	2520	2520	840	840	840	840
10.4 k	3120	3120	1040	1040	1040	1040
13.4 k	4020	4020	1340	1340	1340	1340
15.6 k	4680	4680	1560	1560	1560	1560
20.8 k	6240	6240	2080	2080	2080	2080
25 k	7500	7500	2500	2500	2500	2500

**Table 2:** Deep Learning Models, with the Number of Convolutional Layers and Parameters.

Deep Learning Model	Number of Convolutional Layers	Number of Parameters (Millions)
**Customized CNN**	5	2.70
**VGG-16**	16	138
**VGG-19**	19	143.67
**ResNet-50**	50	23.78
**Inception V3**	48	21.80

**Table 3:** Experimental results of all models.

Models								
Dataset Size	Customized CNN Model							
	Confusion Matrices			Validation Accuracy	Testing Accuracy	Precision	Recall	F Score
2.8 k	Class	Crack (0)	Non-Crack (1)	0.991	0.985	1.000	0.973	0.986
	Crack (0) Non-Crack (1)	297	0					
		8	255					
5.6 k	Crack (0)	530	2	0.981	0.978	0.996	0.960	0.977
	Non-Crack (1)	22	566					
8.4 k	Crack (0)	828	8	0.982	0.980	0.990	0.971	0.981
	Non-Crack (1)	24	820					
10.4 k	Crack (0)	1020	17	0.964	0.952	0.983	0.925	0.953
	Non-Crack (1)	82	961					
13.4 k	Crack (0)	1309	4	0.984	0.958	0.997	0.925	0.959
	Non-Crack (1)	106	1261					
15.6 k	Crack (0)	1568	3	0.975	0.890	0.998	0.822	0.901
	Non-Crack (1)	339	1210					
20.8 k	Crack (0)	2133	5	0.957	0.908	0.997	0.850	0.918
	Non-Crack (1)	374	1648					
25 k	Crack (0)	2449	16	0.967	0.958	0.997	0.850	0.918
	Non-Crack (1)	192	2343					
	VGG-16 Model							
2.8 k	Class	Crack	Non-Crack	0.997	0.998	1.000	0.996	0.998
	Crack (0)	297	0					
	Non-Crack (1)	1	262					
5.6 k	Crack (0)	531	1	0.999	0.999	0.998	1.000	0.999
	Non-Crack (1)	0	588					
8.4 k	Crack (0)	832	4	0.999	0.997	0.995	0.998	0.997
	Non-Crack (1)	1	843					
10.4 k	Crack (0)	1030	7	0.994	0.992	0.993	0.992	0.992
	Non-Crack (1)	8	1035					
13.4 k	Crack (0)	1312	1	0.998	0.998	0.999	0.997	0.998
	Non-Crack (1)	3	1364					
15.6 k	Crack (0)	1555	16	0.997	0.994	0.989	0.998	0.994
	Non-Crack (1)	2	1547					
20.8 k	Crack (0)	2117	21	0.994	0.992	0.990	0.994	0.992
	Non-Crack (1)	11	2011					
25 k	Crack (0)	2450	60	0.987	0.986	0.976	0.996	0.986
	Non-Crack (1)	9	2481					
	VGG-19 Model							
2.8 k	Class	Crack	Non-Crack	0.900	0.899	0.976	0.855	0.911
	Crack (0)	290	7					
	Non-Crack (1)	49	214					
5.6 k	Crack (0)	519	13	0.917	0.916	0.975	0.866	0.917
	Non-Crack (1)	80	508					
8.4 k	Crack (0)	810	26	0.944	0.937	0.968	0.911	0.939
	Non-Crack (1)	79	765					
10.4 k	Crack (0)	1009	28	0.929	0.929	0.973	0.894	0.932
	Non-Crack (1)	119	924					
13.4 k	Crack (0)	1278	35	0.955	0.951	0.973	0.930	0.951
	Non-Crack (1)	95	1272					
15.6 k	Crack (0)	1527	44	0.954	0.951	0.972	0.934	0.952
	Non-Crack (1)	107	1442					
20.8 k	Crack (0)	2068	70	0.952	0.952	0.967	0.941	0.954
	Non-Crack (1)	129	1893					
25 k	Crack (0)	2396	69	0.960	0.960	0.972	0.949	0.960
	Non-Crack (1)	128	2407					
	ResNet-50 Model							
2.8 k	class	Crack	Non-Crack	0.994	0.994	0.988	1.000	0.994
	Crack (0)	260	3					
	Non-Crack (1)	0	297					
5.6 k	Crack (0)	578	10	0.992	0.983	0.983	0.991	0.987
	Non-Crack (1)	8	524					
8.4 k	Crack (0)	823	20	0.994	0.987	0.976	0.998	0.987
	Non-Crack (1)	1	836					
10.4 k	Crack (0)	1027	16	0.990	0.986	0.984	0.987	0.986
	Non-Crack (1)	13	1024					
13.4 k	Crack (0)	1358	9	0.995	0.995	0.993	0.998	0.996
	Non-Crack (1)	2	1311					
15.6 k	Crack (0)	1526	23	0.990	0.990	0.985	0.996	0.990
	Non-Crack (1)	6	1565					
20.8 k	Crack (0)	1985	37	0.990	0.988	0.981	0.995	0.988
	Non-Crack (1)	10	2128					
25 k	Crack (0)	2433	369	0.994	0.994	0.984	0.991	0.987
	Non-Crack (1)	50	2148					
	Inception V3 Model							
2.8 k	class	Crack	Non-Crack	0.996	0.973	0.943	1.000	0.970
	Crack (0)	248	15					
	Non-Crack (1)	0	297					
5.6 k	Crack (0)	588	0	0.998	0.952	1.000	0.931	0.964
	Non-Crack (1)	53	479					
8.4 k	Crack (0)	838	5	0.995	0.994	0.994	0.994	0.994
	Non-Crack (1)	5	832					
10.4 k	Crack (0)	1031	12	0.990	0.987	0.988	0.986	0.987
	Non-Crack (1)	14	1023					
13.4 k	Crack (0)	1288	79	0.997	0.970	0.942	1.000	0.970
	Non-Crack (1)	0	1313					
15.6 k	Crack (0)	691	858	0.991	0.725	0.446	1.000	0.617
	Non-Crack (1)	0	1571					
20.8 k	Crack (0)	1622	400	0.979	0.899	0.802	0.987	0.885
	Non-Crack (1)	20	2118					
25 k	Crack (0)	2463	71	0.985	0.982	0.972	0.992	0.982
	Non-Crack (1)	18	2448					

**Table 4:** Accuracy of the models at the 1st and 20th epoch.

Dataset Size	Models									
	CNN Model		VGG-16		VGG-19		ResNet-50		Inception v3	
	Accuracy									
	1st	20th	1st	20th	1st	20th	1st	20th	1st	20th
2.8 k	0.976	0.983	0.980	0.998	0.894	0.900	0.673	0.954	0.976	0.973
5.6 k	0.958	0.970	0.965	0.996	0.867	0.917	0.475	0.983	0.975	0.952
8.4 k	0.963	0.977	0.969	0.994	0.944	0.937	0.498	0.987	0.972	0.994
10.4 k	0.935	0.933	0.968	0.990	0.857	0.929	0.550	0.986	0.957	0.987
13.4 k	0.977	0.980	0.992	0.989	0.898	0.951	0.935	0.995	0.957	0.970
15.6 k	0.962	0.899	0.984	0.995	0.952	0.951	0.980	0.990	0.936	0.725
20.8 k	0.942	0.937	0.975	0.993	0.926	0.952	0.984	0.988	0.970	0.899
25 k	0.946	0.941	0.982	0.986	0.9412	0.960	0.980	0.994	0.977	0.982

**Table 5:** Comparison of models computational time and size.

Model	Patch Size	Single Patch Computation Time (Seconds)	Total Image (2240 × 2240) Computation Time (Seconds)	Model Size
Customized CNN Model	224 × 224	0.0048	0.48	10.3 MB
VGG-16 Model [36]	224 × 224	0.1995	19.95	528 MB
VGG-19 Model [36]	224 × 224	0.2093	20.93	549 MB
ResNet-50 Model [37]	224 × 224	0.0662	6.62	98 MB
Inception-V3 Model [38]	229 × 229	0.0385	3.85	92 MB

**Table 6:** Comparison of the proposed work with other CNN works.

Reference	Dataset	No. of Conv Layers	No. of Fully Connected Layers	No. of Epochs	No. of Images	Accuracy	Precision	Recall	F1 Score
Zhang et al. [26]	CCIC [47]	4	2	<20	1000 k	NA	0.8696	0.9251	0.8965
Sattar et al. [46]	SDNET [46]	5	3	B = 32 W = 30 P = 30	56 k	B = 0.9045 W = 0.8745 P = 0. 9486	NA	NA	NA
Sattar et al. [56]	SDNET [46]	5	3	30	18 k	0.97	NA	NA	0.80
Słoński et al. [54]	SDNET [46]	4	3	100	5.2 k	0.85	NA	NA	NA
Fang et al. [55]	CCIC [47] +SDNET [46] + Dataset from [56]	3	2	20	184 k	NA	0.184	0.943	0.307
Proposed Method	CCIC [47] +SDNET [46]	4	2	20	25 k	0.967	0.997	0.850	0.918

NA = Not Available, B = Bridge, W = Wall, P = Pavement, Conv = Convolutional, FRCNN = Faster Recurrent CNN.

## Data Availability

Not applicable.
